# Regulatory frameworks involved in the floral induction, formation and developmental programming of woody horticultural plants: a case study on blueberries

**DOI:** 10.3389/fpls.2024.1336892

**Published:** 2024-02-12

**Authors:** Guo-qing Song, Zongrang Liu, Gan-yuan Zhong

**Affiliations:** ^1^ Plant Biotechnology Resource and Outreach Center, Department of Horticulture, Michigan State University, East Lansing, MI, United States; ^2^ USDA Agricultural Research Services, Appalachian Fruit Research Station, Kearneysville, WV, United States; ^3^ USDA Agricultural Research Services, Grape Genetics Research Unit and Plant Genetic Resources Unit, Geneva, NY, United States

**Keywords:** chilling requirement, deciduous plant, florigen, floral activation, floral initiation, flowering mechanism, woody plant

## Abstract

Flowering represents a crucial stage in the life cycles of plants. Ensuring strong and consistent flowering is vital for maintaining crop production amidst the challenges presented by climate change. In this review, we summarized key recent efforts aimed at unraveling the complexities of plant flowering through genetic, genomic, physiological, and biochemical studies in woody species, with a special focus on the genetic control of floral initiation and activation in woody horticultural species. Key topics covered in the review include major flowering pathway genes in deciduous woody plants, regulation of the phase transition from juvenile to adult stage, the roles of *CONSTANS* (*CO*) and *CO*-like gene and *FLOWERING LOCUS T* genes in flower induction, the floral regulatory role of GA-DELLA pathway, and the multifunctional roles of MADS-box genes in flowering and dormancy release triggered by chilling. Based on our own research work in blueberries, we highlighted the central roles played by two key flowering pathway genes, *FLOWERING LOCUS T* and *SUPPRESSOR OF OVEREXPRESSION OF CONSTANS 1*, which regulate floral initiation and activation (dormancy release), respectively. Collectively, our survey shows both the conserved and diverse aspects of the flowering pathway in annual and woody plants, providing insights into the potential molecular mechanisms governing woody plants. This paves the way for enhancing the resilience and productivity of fruit-bearing crops in the face of changing climatic conditions, all through the perspective of genetic interventions.

## 1 Introduction

Flowering represents a vital phase in the reproductive developmental of plants, ultimately resulting in the generation of seeds for subsequent generations. In agriculture, a robust flowering process, encompassing floral induction, formation, and developmental programming, stands as a fundamental prerequisite for achieving productive crop cultivation. The persistent trend of global warming, coupled with burgeoning populations and the depletion of natural resources, has presented significant challenges to agricultural production. For staple crops, warming can curtail agricultural output by shifting optimal growth zones and/or diminishing both cropping frequency and yields (Zhu et al., 2022). In the case of woody fruit crops, particularly temperate fruit trees, the impacts of global warming are profound, exerting adverse effects on floral development, dormancy release, and fruit growth (Luedeling et al., 2011). A pertinent instance is the scenario wherein insufficient chilling, triggered by climatic shifts, precipitates decreased bud break and reduced flower quality, leading to a reduction in fruit production (Atkinson et al., 2013).

Annual plants have evolved to respond to seasonal variations, facilitating a seamless transition from vegetative to reproductive phases. Extensive investigations using the model plant Arabidopsis (*Arabidopsis thaliana*) and cereal crops have yielded a wealth of valuable insights. These studies have unveiled pivotal regulatory nodes governing floral initiation and flowering time, encompassing pathways tied to aging, photoperiod, autonomous/vernalization, and gibberellin stimuli (see reviews by Greenup et al., 2009; Fornara et al., 2010; Andres and Coupland, 2012; Conti, 2017; Kinoshita and Richter, 2020; Izawa, 2021; Liu et al., 2021). At the center of this regulatory matrix stand two major integrators: *FLOWERING LOCUS T* (*FT*) and *SUPPRESSOR OF OVEREXPRESSION OF CONSTAN 1* (*SOC1*). *FT* exerts a positive influence on *SOC1*, with this FT-to-SOC1 module occupying a central role in plant flowering, exhibiting evolutionary conservation across diverse plant species (Fornara et al., 2010; Lee and Lee, 2010). In Arabidopsis, *FT* emerges as a direct downstream target of both *CONSTANS* (*CO*) within the photoperiod pathway and *FLOWERING LOCUS C* (*FLC*) in the vernalization/autonomous pathway. *SOC1*, on the other hand, is under the direct sway of *SQUAMOSA PROMOTER BINDING PROTEIN-LIKE* (*SPL*) in the aging pathway, alongside FLC and DELLA proteins within the gibberellin pathway (Wang et al., 2009; Preston and Hileman, 2013; Bao et al., 2020).

The elucidation of the intricate gene networks underpinning each flowering pathway in Arabidopsis has laid a foundational framework, setting the stage for analogous insights into the flowering mechanisms of other plants. In this review, our attention is directed towards the intricacies of flowering mechanisms in woody plants, with a particular focus on fruit-bearing crops. We provide a concise summary of recent advancements and present a gene network that explains possible flowering mechanism in woody plants, specifically focusing on deciduous temperate fruit crops. Within this framework, we underscore the role of *FT* in floral induction and *SOC1*’s involvement in floral programming. This gene-centric network not only deepens our understanding of flowering mechanisms in woody plants but also serves as a valuable knowledge resource for orchard management for fruit growers.

## 2 Major flowering pathway genes in deciduous woody plants

**Figure 1:**
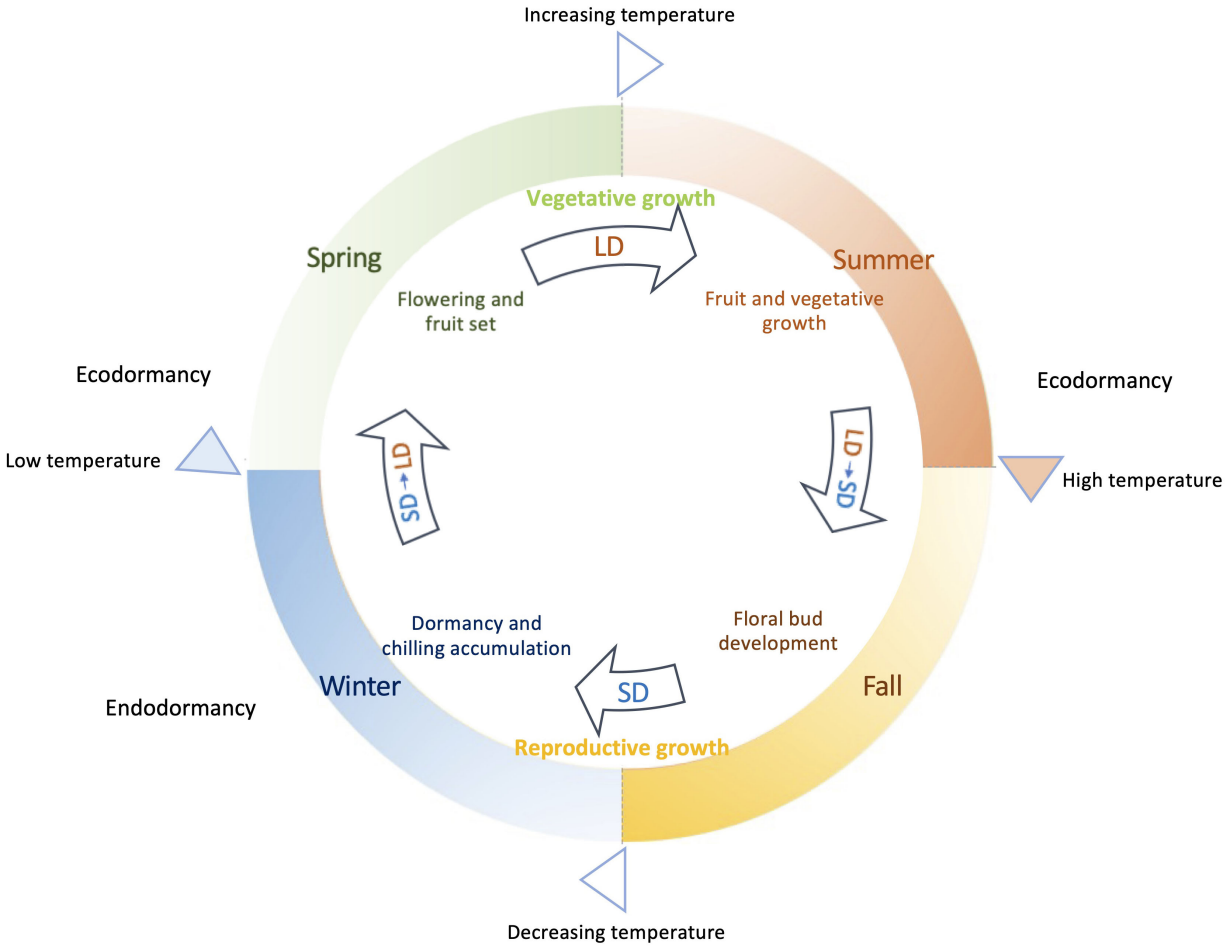
Annual growth cycle of deciduous woody plants guided by seasonal daylight and temperatures. Plant flowering, fruiting, and vegetative growth mainly occur in spring and summer, when it is in long day (LD) conditions with growing daylength associated with increasing/higher average temperatures. Growth cessation and dormancy happen in fall and winter under decreasing temperature and mostly short day (SD) conditions, when flower bud formation is achieved and endodormancy is induced. Enough chilling accumulation is needed before sufficient warm accumulation breaks endodormancy in spring. Low temperatures or insufficient warm accumulation causes ecodormancy in spring for those fully chilled buds. Extreme high temperatures in summer can result in ecodormancy, during which plant growth is temporarily arrested.

The process of flowering in deciduous woody plants is a product of intricate genetic and environmental influences and their interactions in responding to the seasonal changes (**Figure 1**). The phases of flowering, fruiting, and vegetative growth predominantly unfold mainly during spring and summer, coinciding with the prevalence of elevated average temperatures. Meanwhile new floral buds for many deciduous woody plants are initiated in the summer period and continuously undergo development through fall. In contrast, winter mark the completion of reproductive growth, onset of growth cessation, and the establishment of dormancy in general. During this period, declining temperatures and shortening day period act as key environmental signals to trigger and drive these biological events. It is generally within these colder months that the development of flower buds occurs and the initiation of endodormancy is likely signaled.

A pivotal facet that distinguishes deciduous woody plants from the vernalization/autonomous pathway observed in Arabidopsis lies in the flowering mechanism driven by chilling accumulation, termed chilling requirement (CR). This accumulation of chilling hours occurs before the eventual release of endodormancy, as temperatures start to rise in spring. This CR-mediated process imparts a unique rhythm to the flowering behavior of deciduous woody plants, in contrast to the continuous floral initiation and flowering progression exhibited by vernalized Arabidopsis plants. Deciduous woody plants often have two distinct phases emerge: the initiation of floral buds mostly during the autumn and early winter, followed by the actual blossoming of these buds in spring subsequent to CR fulfillment (**Figure 1**).

Flower regulation in deciduous fruit trees is distinct from annuals. In annuals, flower initiation begins with the transition from vegetative to inflorescence stage and flower formation and development are typically completed within a single season (see reviews by Baurle and Dean, 2006; Irish, 2010). On the other hand, flowers from seedling-derived fruit trees can only be initiated and developed after the tree reaches adulthood, which can take several years. Even in adult trees, flower initiation and development occur over two growing seasons, not one (Wilkie et al., 2008; Sun et al., 2022). For example, floral bud initiation in apple and peach trees occurs in summer and basic morphological structures such as sepal, petal, stamen, and carpel are developed during the fall before entering a fully dormant state, remaining in a “resting” state during winter and then resumed developmental pace before flowering next spring. Winter chilling is indispensable for driving dormancy out (Arora et al., 2003). It was also found that chilling is essential for driving morphological differentiation within buds during winter (Luna et al., 1990; Luna et al., 1991; Luna et al., 1993; Reinoso et al., 2002a; Reinoso et al., 2002b; Julian et al., 2011). In fact, the formation of specific floral tissue in response to chilling is a morphological indicator that the floral buds are out of dormant state and have entered an ecodormant state capable of responding to warm stimuli, resuming developmental pace and achieving reproduction success (Wang et al., 2004; Wang et al., 2016). Notably, floral initiation, formation and development are regulated by endogenous physiological states and seasonal thermal regimes.

### 2.1 Regulation of the phase transition from juvenile to adult stage

**Figure 2:**
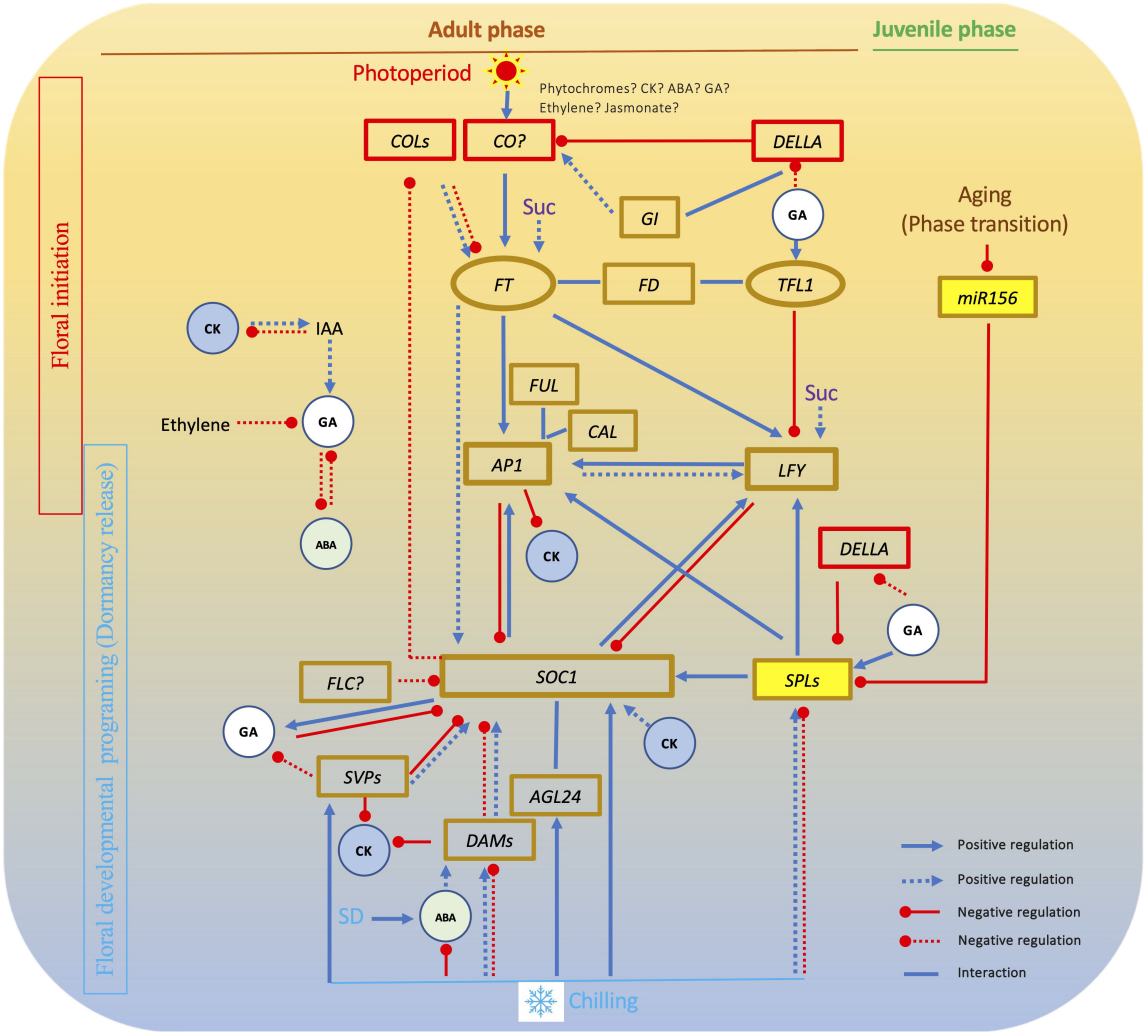
Interactions among key flowering pathway genes in chilling-dependent woody plants. In this gene network, *FT* plays an essential role in floral initiation and formation. *SOC1* has a central role in floral activation by interacting with other MADS box genes. GA affects both floral initiation and floral activation. Other hormones and sucrose have impact on floral initiation or floral activation. Solid lines show relationships revealed in *Arabidopsis*. Dot lines shows relationships found in woody plants. SD, short day; GA, gibberellin; Suc, sucrose; CK, cytokinin; ABA, abscisic acid; IAA, Indole-3-acetic acid.

In the genetic realm, the majority of woody fruit crops exhibit a juvenile phase spanning from days to years, during which seedlings remain incapable of flowering even when subjected to suitable environmental triggers (Samach, 2012). In *Arabidopsis*, the transition from vegetative to inflorescence meristem appears to be regulated by the aging pathway requiring a miR156 (microRNA156) that controls *PROMOTER BINDING PROTEIN-LIKE* genes *(SPLs)* (Wang et al., 2009; Wu et al., 2009; Teotia and Tang, 2015; Xu et al., 2016b; Hyun et al., 2017). In this pathway, miR156 negatively regulates the activity of flowering activator *SPLs*. This family of *SPLs*, known for their multifunctionality, catalyzes floral transition by augmenting the expression of key genes such as *LEAFY* (*LFY*) and MADS box genes *SOC1* and *AP1* (Albani and Coupland, 2010; Lee and Lee, 2010; Ma et al., 2021). The engagement of the miR156-SPL module in the transition from juvenile to adult phases has been validated in various plants, including apple (Zhang et al., 2015a; Jia et al., 2017; Zheng et al., 2019), the conserved nature of this miR156-SPL module within the aging pathway is acknowledged across annual and perennial plant species. Yet, substantiating this module’s presence in other woody plants other than apple remains a necessity, along with addressing the intriguing query surrounding the divergent durations of juvenile phases observed among different woody plant species (Huijser and Schmid, 2011; Morea et al., 2016) (**Figure 2**).

Of notable significance is the involvement of the DELLA proteins that act as master regulators that rewire a multitude of transcriptional networks to control diverse biological responses (Briones-Moreno et al., 2023). One of the Arabidopsis DELLA factors exhibits a propensity to interact with distinct *SPLs*, yielding disparate outcomes. These interactions can either spark the initiation of floral primordia, such as through the activation of *AP1* transcription via binding with *SPL9*, or act to inhibit *SPL* function, thus serving as a brake on the flowering process. This dual nature confers upon interactions of gibberellin (GA) and DELLA proteins to produce varying effects on the reproductive journey, depending on the developmental stage, all orchestrated by the intricate behavior of DELLA proteins (Yu et al., 2012; Yamaguchi et al., 2014; Bao et al., 2020).

The phase transition in fruit trees is also regulated by *TFL1* and its related genes. For example, when the *TFL1* gene is knocked out or down in apple plantlets, the flower inhibition imposed by juvenility is erased, allowing for flowering in re-juvenilized shoots as quickly as a few months, instead of the usual 3-6 years (Kotoda et al., 2006; Charrier et al., 2019). However, it is not yet clear how the miR156-*SPL* pathway interacts with *TFL1*.

### 2.2 The roles of *CONSTANS*- and *CONSTANS*-*LIKE* genes-*FT* regulatory framework in flower induction in fruit trees

Photoperiod, notably the duration of daylight, constitutes a pivotal environmental cue orchestrating plant flowering. This process elucidates the photoperiod pathway, wherein the *CO* gene plays a crucial role in transducing light signals to regulate *FT* expression (Samach et al., 2000; Kinoshita and Richter, 2020). While the CO-FT module has been unequivocally demonstrated in numerous annual plants, suggesting its theoretical conservation across all species (Putterill et al., 1995; Yano et al., 2000; Griffiths et al., 2003; Kim et al., 2008; Samach, 2012; Song et al., 2012; Lymperopoulos et al., 2018; Bao et al., 2020), the centrality of *COLs* (*CO-LIKE* genes) in the orchestration of flowering time remains a subject of contention. This is attributed in part to the intricate diversity in characterizing the *COL* gene family (Wong et al., 2014). Ectopic expression analyses of peach *CO* in Arabidopsis have indirectly supported the conservation of the CO-FT module in trees, hinting at a broader applicability (Böhlenius et al., 2006; Zhang et al., 2015b). In the case of apple, distinctive expression patterns of apple *COLs* in comparison to Arabidopsis genes suggest a divergent CO-FT module (Jeong et al., 1999). Expression studies have brought to light varying facets: grape *CO* linked to flowering initiation and *COL1* associated with dormancy (Almada et al., 2009); six pear (*Pyrus bretschneideri*) *COLs*, out of a total of 15, showed circadian clock and photoperiod-regulated expression (Wang et al., 2017); and Mango (*Mangifera indica* L.) *CO* and *COLs* implicated in photoperiod-mediated flowering, with CO-FT conservativity warranting further clarification (Liu et al., 2020a; Liu et al., 2022). Similar investigations have extended to bamboo (*Phyllostachys violascens*), poplar (*Populus trichocarpa*), and Rabbiteye blueberries (*Vaccinium virgatum* Aiton), demonstrating their roles in photoperiod-controlled flowering through expression patterns or Arabidopsis ectopic expression (Xiao et al., 2018; Li et al., 2020; Omori et al., 2022).

However, the conclusive validation of the central role of the CO-FT module in woody plants awaits direct evidence from gain-of-function (*e.g.*, overexpression) or loss-of-function (*e.g.*, gene silencing/knockout) studies. The intricate multifunctional roles of the *COLs* family have added layers of complexity to their functional scrutiny. While the comprehensive understanding remains elusive, the CO-FT involvement in flowering function has yet to be ruled out. Concurrently, the interplay between *CO* expression and light in Arabidopsis reveals the intricate dance of phytochrome-phytohormone interactions in regulating flowering time and broader aspects of plant growth and development (Fornara et al., 2010; Lymperopoulos et al., 2018). The regulatory role of DELLAs in the GA pathway as a negative regulator of *CO* expression, along with the indirect influences of abscisic acid (ABA) and Jasmonate, further accentuates the intricate tapestry of these regulatory networks (Davis, 2009; Tiwari et al., 2010; Xu et al., 2016a; Bao et al., 2020; Izawa, 2021; Serrano-Bueno et al., 2021). Flower initiation in apple, peach, and other trees occurs in summer or late summer, suggesting that unlike in Arabidopsis, photoperiod signals may not play a role in flower induction. Instead, other endogenous signals such as physiological state, hormone homeostasis, or nutrient balance (*e.g*., photosynthetic output) might be influencing flower formation. As a result, the CO-FT module could gain new functions and interact with these endogenous signals in fruit trees to control floral initiation.

### 2.3 The opposite floral regulatory roles of GA-DELLA pathway in annuals and woody fruit trees

In Arabidopsis, phytohormones known as GAs play substantial roles in various aspects of plant growth and development, spanning processes such as seed germination, elongation growth, and the regulation of flowering time (Michniewicz and Lang, 1962; Yamauchi et al., 2004; Willige et al., 2007; Yamaguchi et al., 2014; Ni et al., 2015). This influence is often mediated through intricate interactions with multiple developmental pathways, facilitated by the DELLA domain which functions as a receiver domain for activated GA receptors (Yamauchi et al., 2004; Willige et al., 2007; Yamaguchi et al., 2014; Ni et al., 2015). The impact of GA on flowering time, whether promoting or inhibiting, hinges on the specific plant species and developmental stages at play (Koshita et al., 1999; Goldberg-Moeller et al., 2013; Pearce et al., 2013; Izawa, 2021). Broadly speaking, GAs tend to exert flowering promotion in long-day and biennial plants, while adopting an inhibitory role in other plant categories, encompassing fruit trees such as citrus (*Citrus reticulata* Blanco × *Citrus temple* Hort. ex Y. Tanaka) and grape (Boss and Thomas, 2002; Goldberg-Moeller et al., 2013; Li-Mallet et al., 2016; Zhang et al., 2019). Nonetheless, this species- and genotype-dependent function of GA in the intricate relationship of flowering introduces a level of uncertainty and complexity, thereby casting a degree of doubt upon GAs as strong candidates for the role of florigen. This sentiment persists despite findings in the grass species *Lolium temulentum*, where specific GAs (GA5 and GA6) emerge as an alternative source of florigenic signal distinct from *FT*, another floral signal originating from leaves (King and Evans, 2003; King et al., 2006).

However, the roles of GAs in regulation of floral formation in perennial flowering species appears to be quite different from that in annual species such as Arabidopsis. GA is thought to be inhibitory to flowering in perennials (Khan et al., 2014), as exemplified by the fact that exogenous applications of GA in citrus, apple and grapevine have been shown to inhibit flower production (Mullins, 1968; Srinivasan and Mullins, 1978; Zhu et al., 2008; Guo et al., 2018). This suggests that GA-generated/stimulated signals are rewired to distinct transcriptional pathways in perennials and annuals, resulting in opposite regulatory outputs. This finding is supported by the gain-of-function mutation of DELLAs, a main target of GA, in annual *Arabidopsis* and perennial grape, which led to repression and promotion of flowering, respectively (Peng and Harberd, 1997; Dill and Sun, 2001; Silverstone et al., 2001; Fleck and Harberd, 2002; Boss et al., 2006). Thus, DELLAs may be rewiring the same GA signals to transcriptional circuits or modules that have opposing functions, or targeting different factors that regulate flowering, resulting in contrasting flowering phenotypes.

### 2.4 Chilling-driven floral development in deciduous fruit trees

Within Arabidopsis, a collection of seven flowering-promoting genes situated within the autonomous pathway assumes a counteractive role against the MADS box gene *FLC*, which stands as a pivotal arbiter of flowering time within the vernalization pathway (Michaels and Amasino, 1999; Simpson, 2004; Michaels, 2009). Functioning as a negative regulator, *FLC* exerts control over flowering by suppressing the expression of *FT* and *SOC1*. The phenomenon of vernalization, characterized by prolonged cold exposure, effectively suppresses *FLC* expression, thus paving the way for the initiation of flowering. The involvement of *SHORT VEGETATIVE PHASE* (*SVP*) in this process emerges through its interaction with *FLC*, mirroring the function of *FLC* in response to vernalization by curbing GA biosynthesis (Gregis et al., 2006; Andres et al., 2014). Similarly, the vernalization pathway of wheat contains *VERNALIZATION 2* (*VRN2*), a zinc finger protein that parallels *FLC* in its role within the wheat vernalization pathway. In this context, *VRN1* and *VRN3* function as counterparts to *FT* and the MADS box gene *AP1*, respectively (Gendall et al., 2001; Yan et al., 2003; Yan et al., 2004; Yan et al., 2006; Woods et al., 2016). It is of note that cereals harbor genes akin to *FLC*, although their functionalities largely remain enigmatic (Kennedy and Geuten, 2020). Collectively, this FLC-regulated vernalization pathway is generally presumed to be conserved in certain plant species, exemplified by Arabidopsis, while displaying evolutionary divergence in others, as seen in the case of cereals (Sheldon et al., 2000; Tadege et al., 2001; Alexandre and Hennig, 2008; Kennedy and Geuten, 2020).

The concept of “Chilling Requirement” (CR), in contrast to vernalization for transition of vegetative to inflorescence meristem in annual plants, elucidates the necessity for an adequate accumulation of chilling hours, pivotal for breaking dormancy and fostering the flowering process within specific woody plant species (Chouard, 1960; Jewaria et al., 2021). In terms of functionality, the CR-mediated flowering pathway in woody plants mirrors the vernalization pathway observed in annual plants (Brunner et al., 2014). The CR in fruit trees and vernalization in annuals and bi-annuals have different impact on flower regulation. Vernalization is required for transition of vegetative to inflorescence meristem while the CR is mostly for regulation of floral bud development rather initiation or formation. Only grape is exception in which conversion of vegetative anagens to inflorescences requires chilling or chilling promotes this conversion. In this sense. chilling acts as a bioregulator and is obligatory for floral development as exemplified by that warm temperature represses the floral development in dormant floral buds but chilling promotes it. As of present, the CR pathways involving *FLC* or *VRN2* have not been definitively substantiated through both forward and reverse genetics methodologies (**Table 1**). However, insights from transcriptome analyses have unveiled the existence of *FLC*-like or *VRN*-like genes across numerous woody plants, including apple, grape, blueberry (*Vaccinium corymbosum* L.), and kiwifruit (*Actinidia chinensis*) (Diaz-Riquelme et al., 2009; Diaz-Riquelme et al., 2014; Varkonyi-Gasic et al., 2014; Porto et al., 2015; Kumar et al., 2016; Song and Chen, 2018b). Employing a forward genetics approach, the discovery of six interconnected *Dormancy-Associated MADS-Box* genes (*DAMs*) emerged as a hallmark of the CR pathway in the evergrowing mutant of peach. Among these, *DAM5* and *DAM6* were found to act as repressors of bud break, while *DAM4* demonstrated pronounced chilling-induced repressor activity, particularly evident at the level of epigenetic regulation (Bielenberg et al., 2008; Jimenez et al., 2009; Li et al., 2009; Jimenez et al., 2010; Wells et al., 2015; Zhu et al., 2020a; Voogd et al., 2022). These *DAMs* stand as potential analogs to *FLC* or *VRN2*, possibly occupying central roles within the CR pathway (Falavigna et al., 2014; Falavigna et al., 2018; Falavigna et al., 2022). Nonetheless, the deficiency of reverse genetics evidence to confirm the functional role of *DAMs* in peaches stems from technical challenges arising from the absence of an efficient peach transformation system for conducting functional gene analysis. Moreover, the striking sequence similarities between *DAMs*, *SVPs*, and *AGAMOUS-LIKE 24* (*AGL24*) of Arabidopsis, as well as their widespread presence in various woody plants (**Table 1**), adds to the intrigue and complexity of their roles.

**Table 1:** Functional analyses of key flowering pathway genes in woody plants.

Plant	Gene	Expression^a^	Function	Reference
Apple (*Malus* × *domestica* Borkh.)	*MdAP1* (*MdMADS5*)	EX	Promotes Arabidopsis flowering	(Kotoda et al., 2002)
Apple	*CONSTANS (CO)-like* (*COL*), *MdCOL1* and *MdCOL2*	EA	Apple *CO*-like genes are significantly different from the *Arabidopsis* genes.	(Jeong et al., 1999)
Apple	*MdDAMb* and *MdSVPa*	OX	Delays bud break. *SVP* genes might also play a role in floral meristem identity.	(Wu et al., 2017a)
Apple	Three *DAM*s and two *SVPs*	KD	Precocious flowering but normal flower morphology, fertility and fruit development were observed.	(Wu et al., 2021)
Apple	*MdSVPa*, *MdSVPb*, and *MdDAM-*like genes	EA	*MdSVPa* and *MdSVPb* but not MdDAM-like genes complement the early-flowering phenotype of Arabidopsis svp-41	(da Silveira Falavigna et al., 2021)
Apple	*MdDAMa* and *MdDAMc*	EA (qRT-PCR)	*MdDAMa* and *MdDAMc* were correlated with the period of endodormancy.	(Mimida et al., 2015)
Apple	*MdFLC1a*, *MdFLC1b*, and *MdFLC1c* (*MdFLC3*)	EX	*MdFLC3* functions as a floral repressor in *Arabidopsis*.	(Kagaya et al., 2020)
Apple	*MdFT1* and *MdFT2*	*MdFT1*-OX	Precocious flowering in apple	(Kotoda et al., 2010)
Apple	*MdFT1*	EX	Promotes flowering in Arabidopsis and poplar	(Trankner et al., 2010)
Apple	*MdLFY*	OX	The use of *LFY* transgenic apple plants for crosses does not seem to be efficient for accelerating breeding cycles.	(Wada et al., 2002; Flachowsky et al., 2010)
Apple	*MdSOC1*	EA	*MdSOC1a* and *MdSOC1b* is compatible with the formation of MADS complexes containing *MdSOC1a* during endodormancy and ecodormancy, and containing *MdSOC1b* during endodormancy.	(Falavigna et al., 2022)
Apple	*MdTFL1*	KD EX KD KD KD	Promotes flowering in apple Delays flowering in Arabidopsis Promotes flowering in apple Delays flowering in Arabidopsis Promotes tobacco flowering	(Kotoda et al., 2006) (Mimida et al., 2009) (Flachowsky et al., 2012) (Zuo et al., 2021) (Do et al., 2022)
Japanese apricot (*Prunus mume*)	*PmDAM*	EX	*PmDAM6* shows growth inhibitory functions in transgenic poplar.	(Sasaki et al., 2011)
Bamboo (*Phyllostachys violascens*)	*PvCO1*	EX	Delays flowering in Arabidopsis	(Xiao et al., 2018)
Black cherry (*Prunus serotina* Ehrh.)	*PsTFL1*	OX	Delays flowering	(Wang and Pijut, 2013)
Blueberry (*Vaccinium corymbosum* L.)	*VcFT*	OX	Precocious flowering	(Song et al., 2013b)
Blueberry	*VcSOC1*	EX	Promotes flowering in tobacco	(Song and Chen, 2018a)
Blueberry	*VcTFL1*	KO	Promotes flowering	(Omori et al., 2021)
Carrizo citrange (*Citrus sinensis* L. Osbeck × *Poncirus trifoliata* L. Raf.)	*AtAP1*	EX	Promotes citrus flowering	(Pena et al., 2001)
Citrus (*Citrus sinensis* L. Osbeck ‘Washington’.)	*CsAP1* and *CsLFY*	*EX*	Early-flowering in Arabidopsis	(Pena et al., 2001; Pillitteri et al., 2004)
Carrizo citrange	*CsFT*	OX	Early flowering, transported signal through transgrafting	(Soares et al., 2020; Sinn et al., 2021)
Citrus (*Citrus sinensis*)	*CsSOC1-*like	EX	Shortens the time taken to flower in the Arabidopsis wild-type ecotypes Columbia and C24	(Tan and Swain, 2007)
Grape (*Vitis vinifera* L.)	*VvFT*	OX	Overexpression of V*vFT* in somatic grapevine embryos repressed the expression of *VvDAM3-SVP* and *VvDAM4-SVP*.	(Vergara et al., 2021)
Grape (*Vitis vinifera* L.)	*VvFT, VvSOC1 (VvMADS8)*	EX	Hastens flowering in Arabidopsis	(Sreekantan and Thomas, 2006)
Grape (*Vitis labruscana* Bailey × *V. vinifera* L.)	*VvSVP*	EX	Abnormal flower morphology and varying degrees of delayed flowering in Arabidopsis	(Dong et al., 2022)
Grape (*Vitis vinifera* L.)	*VvTFL1*	EX	Delays flowering in tobacco and Arabidopsis	(Boss et al., 2006)
Grape (*Vitis vinifera* L.)	*VvCO* and *VvCOL1*	EA	*VvCO* expression in latent buds is in agreement with a function during flowering induction.	(Almada et al., 2009)
Kiwifruit (*Actinidia chinensis*)	*AcFLC*	KO	*AcFLCL* promotes flowering.	(Voogd et al., 2022)
Kiwifruit	*AcFT*	EX and OX	Induces early flowering in transgenic Arabidopsis. OX results in *in vitro* flowering but the plants are not viable.	(Moss et al., 2018)
Kiwifruit	*AcSOC1* (9)	EX	Promotes flowering in Arabidopsis	(Voogd et al., 2015)
Kiwifruit	*AcSVP3*	*OX*	No effect on vegetative growth, dormancy, or flowering time	(Wu et al., 2014)
Kiwifruit	*AcTFL1*	KO	Promotes flowering	(Varkonyi-Gasic et al., 2019)
Loquat (*Eriobotrya japonica* Lindl.)	*EjAP1*	EX	*EjAP1* can partially complement the *ap1-1* mutant of Arabidopsis.	(Liu et al., 2011; Liu et al., 2013b)
Loquat	*EjLFY*	EX	Early-flowering in Arabidopsis	(Liu et al., 2011)
Loquat	*EjSOC1*	EX	Promotes flowering in Arabidopsis	(Jiang et al., 2019c)
Loquat	*EjSVP*	EX	Overexpression of *EjSVP2* affected the formation of *Arabidopsis thaliana* flower organs.	(Jiang et al., 2019b)
Loquat	*EjTFL1*	EX	Delays flowering in Arabidopsis	(Jiang et al., 2019a; Jiang et al., 2020)
Mango (*Mangifera indica* L.)	*MiCOL* and MiCO	EX	Delays flowering in Arabidopsis	(Liu et al., 2020a; Liu et al., 2022)
Peach (*Prunus persica*)	*PpCO*	*EX*	Restores the late flowering phenotype of the Arabidopsis *co-2* mutant	(Zhang et al., 2015b)
Peach	*PpDAM*	EA	Chilling downregulates DAM1 and DAM3-6 in dormant floral buds.	(Zhu et al., 2020a)
Peach	*PpDAM*	EA	*DAM3*, *DAM5* and *DAM6* were winter expressed. The expression patterns of *DAM5* and *DAM6* are consistent with a role as quantitative repressors of bud break.	(Jimenez et al., 2010)
Peach	*PpFT*	EX	Promotes flowering in Arabidopsis	(Zhang et al., 2015b)
Peach	*PpTFL1*	EX	Delays flowering in Arabidopsis	(Chen et al., 2013)
Pear (*Pyrus pyrifolia* Nakai)	*PypAP1*	EX	Early-flowering in Arabidopsis	(Liu et al., 2013a)
Pear (*Pyrus bretschneideri*)	*PbCOL*	EA	Six *PbCOLs* were found to be regulated by both circadian clock and photoperiod.	(Wang et al., 2017)
Pear (*Pyrus pyrifolia* Nakai)	*PypDAM*	KD	Increases bud break rate	(Gao et al., 2021)
Pear (*Pyrus pyrifolia* Nakai)	*PypDAM1*	EA	*PpDAM1* increases in endodormancy.	(Ubi et al., 2010; Tuan et al., 2017)
Pear (*Pyrus communis* L.)	*PycFT2*	EX	Promotes flowering in tobacco but not in apple	(Freiman et al., 2015)
Pear (*Pyrus communis* L.)	(*Betula pendula*) APETALA1/FRUITFULL MADS-box gene *BpMADS4*	EX	Promotes flowering	(Tomes et al., 2023)
Pear (*Pyrus bretschneideri*)	*PybSOC1*	EX	Leads to early flowering phenotype in *Arabidopsis*	(Liu et al., 2020b)
Pear (*Pyrus pyrifolia* Nakai)	*PypTFL1*	EA	*PpTFL1* is involved in floral induction.	(Bai et al., 2017)
European plum (*Prunus domestica* L.)	*PdoDAM1-6*	EA	*PdoDAM3* & *4* are of a little difference from the others.	(Quesada-Traver et al., 2020)
European plum	Poplar *FT1* isolated from *Populus trichocarpa*	EX	Promotes flowering in plum	(Srinivasan et al., 2012)
Chinese plum (*Prunus salicina* Lindl.)	*PsDAM1-6*	EA	*PsDAM6* expression was repressed by chilling treatment	(Fang et al., 2022)
Poplar (*Populus trichocarpa*)	*PtFT1*	EX *PtFT1* inducible expression	Promotes early flowering in *Arabidopsis* Early flowering	(Klocko et al., 2016)
Poplar (*Populus trichocarpa*)	*PtLFY*	OX and EX KD	Accelerates flowering in Arabidopsis. One of the many tested transgenic lines of Populus flowered precociously. Several leaf morphology and productivity traits were statistically and often substantially different in sterile vs. normal flowering RNAi-*LFY* trees.	(Rottmann et al., 2000) (Klocko et al., 2021)
Hybrid poplar (*Populus tremula* × *alba)*	*SOC1* (*MADS12*	*OX*	*Promotes bud break in ecodormant poplars*	(Gómez-Soto et al., 2021)
Poplar (*Populus trichocarpa*) Hybrid poplar (*Populus tremula* × *alba)* Hybrid poplar (*P. tremula x tremuloides*)	*SVP*-like from *Populus trichocarpa*	OX	Delay the onset of flowering	(Goralogia et al., 2021)
Poplars (*Populus* spp.)	*PopCEN1 (TFL1)*	KD	None of the transgenics exhibited flowering or other obvious phenotypic effects	(Mohamed et al., 2010)
Rose (*Rosa chinensis*)	*RcAP1*	EX	Early-flowering in *Arabidopsis*	(Han et al., 2019)
Rose	*RoKSN*, a TFL1 homologue	EX	Leads to the absence of flowering in *Arabidopsis*	(Randoux et al., 2014)
Sweet cherry (*Prunus avium*)	*PaAP1*	EX	An early flowering in *Arabidopsis*	(Wang et al., 2013)
Sweet cherry	*PavDAM1* and *PavDAM5*	EX	Results in plants with abnormal flower and seed development in Arabidopsis	(Wang et al., 2020)
Sweet cherry	*PavDAM*	EX	Results in plants with abnormal flower and seed development in Arabidopsis	(Branchereau et al., 2022)
Sweet cherry	*PavMADS1* and *PavMADS2* (DAM)	KD	Silencing of *PavMADS1* and *PavMADS2* coincided with an increase in *FT* expression during dormancy	(Rothkegel et al., 2017)
Sweet cherry	*PavFT*	EX	Promotes flowering in Arabidopsis	(Yarur et al., 2016)
Sweet cherry	*PavSVP and PavSVPL*	EX	Delays flowering and floral defects phenotype in *Arabidopsis*	(Wang et al., 2021)

**^a^** Overexpression (OX): constitutive expression of a gene from the same species/genotype. Ectopic expression (EX): constitutive expression of gene from different species. Expression analysis (EA): expression analysis by RNA sequencing or quantitative reverse transcript PCR (qRT-PCR). Gene knockdown (KD): repression of gene expression using RNAi or antisense expression. Gene knockout (KO): completely inhibit gene expression by removing the gene using gene editing.

Emerging as pivotal contenders within the CR pathway, both *DAMs* and *FLC*-like genes have extensively been studied across various significant woody fruit crops through techniques such as expression analysis, ectopic expression, overexpression, gene silencing, and gene knockout (**Table 1**). These comprehensive investigations have revealed several key insights:

1) The diversity and prevalence of *DAMs* and *FLC*-like genes present in woody plants.
2) Their integral involvement in flowering orchestrated by chilling exposure, while also revealing the nuanced role these genes play, often dependent on the specific species and genotype.
3) Contrary to the well-defined centrality of *FLC* in Arabidopsis or *VRN2* in cereals within their respective vernalization pathways, no singular *DAM* or *FLC*-like gene in woody fruit crops appears to assume a universally conserved and central role in the CR pathway.

In fact, for all plant species requiring either vernalization or chilling, the pivotal factor in regulating flowering time is not individual genes like *FLC* in Arabidopsis, *VRN1* in cereals, or *DAMs* (*SVPs* or *AGL24*), but rather the entire MADS-box gene family.

## 3 *FT*-dominated floral induction and *SOC1*-centered floral activation in deciduous woody plants

As elucidated earlier, the five well-established pathways in Arabidopsis—namely age, photoperiod, GA, autonomous, and vernalization—stand as the benchmark for unraveling flowering mechanisms in diverse plant species. The wealth of insights garnered through analyses of flowering pathways in myriad other plants has yielded a plethora of evidence. This evidence aids in discerning both conserved and nonconserved genes and intricate networks governing plant flowering. This accumulation of knowledge paves the way for endeavors aimed at manipulating individual gene(s) to regulate flowering time and enhance yields. Notably, it stands to reason that plants, including woody varieties, have, to varying degrees, evolved distinct flowering pathways. Drawing from the available literature, it becomes evident that at the core of floral induction resides FT-centered processes, while SOC1-centered mechanisms prevail in orchestrating floral activation across a wide spectrum of plants, if not universally so (**Figure 2**).

### 3.1 *FT*-dominated floral induction

FT acts as a critical integrator, assimilating signals for floral transition from approximately 10 activators and 30 repressors largely stemming from photoperiod and vernalization pathways, thus instigating flowering in Arabidopsis (Kobayashi et al., 1999; Wigge et al., 2005; Pin and Nilsson, 2012; Kinoshita and Richter, 2020; Liu et al., 2021). It stands prominently poised as a top contender for the florigen role (Turck et al., 2008; Turnbull, 2011; Pin and Nilsson, 2012). On the converse, *TERMINAL FLOWER 1* (*TFL1*), a homolog of *FT*, exerts an opposing effect within Arabidopsis (Bradley et al., 1997; Kobayashi et al., 1999). Within the *FT/TFL1* gene family, an assemblage of six members comes into view, encompassing *FT*, *TWIN SISTER OF FT*, *TFL1*, *BROTHER OF FT AND TFL1* (*BFT*), *MOTHER OF FT AND TFL1* (*MFT*), and *ARABIDOPSIS THALIANA CENTROADIALIS HOMOLOGUE* (*ATC*) (Yoo et al., 2010; Ryu et al., 2011; Liu et al., 2016). *FT* and *TFL1* function through direct interactions with the bZIP transcription factor *FD*. The constitutive upregulation of *FT* or its orthologs (hereafter FT-CX) expression aligns with a proclivity for flowering promotion. Conversely, the persistent elevation of *TFL1* or its orthologs (hereafter TFL1-CX) tends to elongate the flowering process. This dualistic phenomenon has been empirically validated across a range of plant species, including select woody plants (**Table 1**). Collectively, mounting evidence gleaned from ectopic expression and overexpression studies substantiates the conservation of *FT* and its orthologs across diverse plants, underscoring their ubiquitous roles as primary inducers within the flowering transition (Pin and Nilsson, 2012; Kinoshita and Richter, 2020; Liu et al., 2021).

The advancement of flowering in woody plants has been effectively catalyzed through the upregulation of FT-CX, as illustrated in **Table 1**. Noteworthy examples in deciduous fruit crops include investigations related to a blueberry FT gene, denoted as *VcFT* (Vc: *Vaccinium corymbosum*), and a poplar (*Populus trichocarpa*) FT1 gene (*PtFT1*) in European plum (*Prunus domestica*). PtFT1-CX induced continuous flowering as demonstrated by Srinivasan et al. (2012). Rigorous exploration has led to findings wherein the constitutive expression of *VcFT* (VcFT-CX) leads to a noteworthy shift in the flowering paradigm. Specifically, this alteration is characterized by the partial reversal of chilling requirements and the induction of early flowering within apical shoot meristems. In transgenic blueberry, this phenomenon resulted in the formation of multiple flower buds at each node, diverging from the single bud occurrence observed in their nontransgenic counterparts (Song et al., 2013b; Walworth et al., 2016). It is noteworthy, tough, that while VcFT-CX exerted significant influence, its effects were not fully comprehensive in substituting the requirement for chilling. Under conditions devoid of sufficient chilling hours, nearly 50% of flower buds failed to attain the requisite potential for blooming (Walworth et al., 2016). Therefore, while VcFT-CX did show phenotypic outcomes including expedited flowering and heightened floral bud formation, it remained inadequate in replicating the roles of chilling requirements intrinsic to blueberry flowering. Notably, the trend of expedited flowering attributed to the constitutive expression of *FT* orthologs and the contrasting delay occasioned by TFL1-CX has been extensively documented across an expanding array of woody plants. Despite this, further investigation is necessary to reveal the intricate involvement of FT in the context of CR-mediated flowering, a domain ripe for exploration (**Table 1**).

*FT* exhibits versatile functionality. Evident from previous research, the overexpression of *FT* orthologs in woody plants such as kiwifruit *FT* (*AcFT*) and VcFT-CX resulted in premature flowering within *in vitro* transformed shoots. However, this effect proved to be potentially overwhelming, hampering the shoots from evolving into viable plants (Moss et al., 2018; Song et al., 2019). The impact of FT-CX at transcript levels becomes readily apparent through comprehensive RNA sequencing analysis. Notably, instances like VcFT-CX provide insight into its extensive impact, significantly elevating *VcFT* expression in both leaves and flower buds; intriguingly, this upregulation was notably absent in roots. Simultaneously, thousands of differentially expressed genes (DEGs) attributed to *VcFT* expression varied across different tissues and developmental stages, even within the same tissue (Walworth et al., 2016; Song et al., 2019; Song et al., 2023).

When scrutinizing major blueberry flowering pathway genes including *VcSOC1*, *VcAP1*, *VcFUL*, *VcLFY*, *VcSPLs*, and *VcSVP* across three distinct tissues, intriguing patterns emerge:

1) In the apical shoot meristems where VcFT-CX induces early flowering, its influence extends to the upregulation of *VcSOC1*, *VcAP1*, *VcFUL*, *VcLFY*, and *VcSPLs* (Walworth et al., 2016).
2) In the mature leaves along the one-year-old shoot, where VcFT-CX results in the emergence of non-blooming floral buds, *VcAP1* and *VcFUL* experience upregulation, while *VcSOC1* and *VcSVP* are repressed (Walworth et al., 2016).
3) Within nonchilled VcFT-CX buds, *VcLFY* expression is elevated, whereas *VcFUL*, *VcSOC1*, and *VcSVP* experience repression (Walworth et al., 2016).
4) When considering VcFT-CX influence in roots, *VcFUL* and *VcSPLs* encounter increased expression, while *VcSOC1* and *VcSVP* are repressed (Song et al., 2019).

These intricate observations collectively suggest that VcFT-CX induces signals for floral bud formation, at least partly through the upregulation of *VcAP1* and *VcFUL* in leaves. Additionally, the expressions of *VcSOC1* and *VcSVP* appear pivotal in determining the timing of both developing and mature floral bud break. The promotion of flowering by *AP1* in various woody plants supports these findings (see **Table 1**). Counter to the flowering promotion led by VcFT-CX, the functional opposite, *VcTFL1*, triggers flowering delay (Omori et al., 2020; Omori et al., 2021; Omori et al., 2022). Intriguingly, VcFT-CX resulted in a reduction in *VcTFL1* expression within young leaves (Walworth et al., 2016). Drawing parallels from Arabidopsis, where *FT* competes with *TFLs* for *FD* binding (Hanano and Goto, 2011; Zhu et al., 2020b). VcFT-CX led to a surprising decrease in *VcFD* expression within nonchilled flower buds. This observation underscores the likelihood of an interaction between *VcFT* and *VcFD* within floral buds, indicating their interplay.

The hereditary promotion of flowering through FT-CX has been substantiated within both self- and cross-pollinated *Eucalyptus* seedlings (Klocko et al., 2016). Conversely, in poplar, the constitutive expression of *LFY*, *AP1*, and *CO* resulted in marginal to negligible advancements in early flowering, a contrast to the robust effect observed with FT-CX (Rottmann et al., 2000; Zhang et al., 2010; Klocko et al., 2016). Similarly, the hereditary transmission of VcFT-CX translated to a remarkable reduction in flowering time for cross-pollinated, transgenic blueberry seedlings, swiftly transitioning them to bloom within a few months, in comparison to the 2-3 years characteristic of their nontransgenic counterparts (Our unpublished data). Clearly, FT-CX emerges as a potent factor in accelerating the transition from the juvenile phase.

*FT* has consistently remained a prominent candidate in the pursuit of identifying the elusive florigen. FT originates within leaves and subsequently moves to the meristems (Turck et al., 2008; Fornara et al., 2010; Krzymuski et al., 2015). Intriguing insights have indicated from transgrafting experiments involving FT-CX materials, underscoring the role of FT-CX in signaling the onset of flowering. In several instances, the FT-CX generated within transgenic leaves, functioning as either a direct or an indirect florigenic signal, exhibited the remarkable capacity to promote flowering in nontransgenic scions through long-distance transportation (Ye et al., 2014; Song et al., 2019; Wu et al., 2022). This phenomenon diverges distinctly from parallel transgrafting studies where FT-CX produced in transgenic roots and stems (in the absence of transgenic leaves) failed to incite flowering in nontransgenic scions (Zhang et al., 2010; Srinivasan et al., 2012; Wenzel et al., 2013; Bull et al., 2017).

In transgrafted blueberry where the transgenic leaves were retained, the influence of VcFT-CX within the transgenic rootstock precipitated floral bud formation within the shoot tips of nontransgenic scions. However, *VcFT* exhibited negligible alterations, while a cluster of phytohormone genes in nontransgenic scions showed varying expressions (Song et al., 2019). Collectively, the evidence demonstrates the status of *FT* as a universal catalyst for the initiation of floral bud formation and the hastening flowering process. To gain a more comprehensive understanding of the long-range florigenic signals originating from *FT* or FT-CX, whether in the form of FT protein, *FT* mRNA, or other derivatives such as phytohormones, further investigations are needed to unravel this intriguing aspect (Wilkie et al., 2008; Izawa, 2021).

### 3.2 *SOC1*-centered floral activation

SOC1 stands as a central integrator within the flowering pathway (see review by Lee and Lee, 2010). Evidence across various plant species highlights *SOC1*’s role as a ubiquitous accelerator of flowering, underscoring its significance (**Table 1**) (Lee et al., 2004; Lee et al., 2008; Seo et al., 2009; Alter et al., 2016; Han et al., 2021; Song et al., 2021). In Arabidopsis, *SOC1* takes on the role of a coordinator, integrating signals from diverse pathways. These connections include the aging pathway, mediated by *SPL*s, the vernalization/autonomous pathway through *FLC*, the photoperiod pathway involving *FT*, and the *GA* pathway, facilitated by GA (**Figure 2**). Notably, *SOC1*’s influence extends to the activation of the *LFY* gene, a key step in establishing the identity of floral meristems or organs (Lee and Lee, 2010).

*SOC1*, alongside *FLC*, *AP1*, *AGL24*, *SVP*, *FUL*, and *CAL*, represents a cohort of MADS box genes encoding MIKCc type proteins characterized by four conserved domains: MADS (M-), intervening (I-), Keratin-like (K-), and C-terminal (C-) (Gramzow and Theissen, 2010; Gramzow and Theissen, 2015). This array of MADS box genes assumes dual roles, vital both in the context of the ABC model of floral development and in governing the temporal aspects of flowering (Amasino, 2010; Heijmans et al., 2012; Smaczniak et al., 2012; Su et al., 2018). Conventionally, *AP1* and *SOC1* have emerged as accelerators of flowering across diverse plant species. Conversely, *FLC* serves as a repressor, exerting repression on the expression of both *FT* and *SOC1* in Arabidopsis. Within Arabidopsis’ vernalization pathway, the antagonistic action of negative regulators, *FLC* and *SVP*, counters the positive regulators *SOC1* and *AGL24* (Fornara et al., 2010; Lee and Lee, 2010). The investigations on CR-mediated flowering woody plant flowering have identified five groups of MADS box genes, specifically the orthologs of *FLC*, *SOC1*, *SVP*, *AGL24*, and *DAMs* (**Table 1**). Amidst these gene clusters, one consensus emerges: the *SOC1* group, a positive regulator, can steer the course of floral initiation and hasten flowering. Yet, the roles of the remaining four gene groups exhibit divergence across diverse plant species. For instance, while *FLC*-like genes have been identified, the extent of their conserved functions in woody plants remains largely unknown (**Table 1**). Notably, apple’s *FLC*-like genes do not mirror *FLC*’s functions precisely (Porto et al., 2015; Nishiyama et al., 2021). Divergent from expectations, a kiwifruit *FLC*-like variant expedites flowering, in contrast to *FLC*’s recognized role in delaying it (Voogd et al., 2022). Similarly, the constitutive expression of an apple *FLC3* variant accelerates flowering in blueberry (Zong et al., 2019; Kagaya et al., 2020).

In woody plants, the CR is orchestrated by MADS box genes *DAMs*, *AGL24-like* genes, and *SVP-like* genes (*SVLs*), which are prominent genes akin to *FLC* functions albeit the scarcity of reverse genetic substantiation (Zhu et al., 2020a; da Silveira Falavigna et al., 2021; Jewaria et al., 2021). These CR-associated MADS box genes act upstream of *SOC1* orthologs and can steer the course of floral development. With chilling accumulation, *SOC1* orthologs are activated. For instance, the grape, blueberry, and apple exhibit an increase in expression of *SOC1* orthologs in response to the accrual of chilling hours (Hattasch et al., 2008; Song and Chen, 2018b; Kamal et al., 2019). In poplar (*Populus tremula* × *alba*), overexpression of a *SOC1-like* variant leads to bud break (Gómez-Soto et al., 2021; Goralogia et al., 2021). In kiwifruit (*Actinidia delicious*), *SOC1-like* genes potentially influence the duration of dormancy, although their role in the transition to flowering remains inconclusive (Voogd et al., 2015). Genetic investigations have shown a linkage between alleles of *SOC1* orthologs and the chilling requisites in apricot (*Prunus armeniaca* L.) and peach genotypes, underlining a pronounced correlation (Trainin et al., 2013; Halasz et al., 2021). Collectively, it is the expression of *SOC1* orthologs that governs the poised readiness for floral bud break and activation subsequent to fulfilling the chilling requirement in woody plants.

In Arabidopsis, the decreased expression of *SVP* during vernalization sets in motion the activation of *SOC1* (or *SOC1-like* gene), thereby initiating the onset of flowering. However, in woody plants, the involvement of *SVP* homologs and *SVLs* in the flowering process exhibits a spectrum of variance contingent upon the particular *SVP* homologs in play. To illustrate, the *SVP* homologs and *SVL*s in kiwifruit, trifoliate orange (*Poncirus trifoliata* L. Raf.), apple, and sweet cherry (*Prunus avium* L.) play the role of suppressors, effectively suppressing budbreak and the flowering cycle (Gregis et al., 2006; Li et al., 2010; Wu et al., 2017a; Wu et al., 2017b; Wang et al., 2021). Meanwhile, in grapevines, the *SVP* homologs unveil a degree of inconsistency, alternating between acting as promoters or inhibitors of flowering (Diaz-Riquelme et al., 2012; Li-Mallet et al., 2016; Arro et al., 2019; Kamal et al., 2019; Dong et al., 2022).

## 4 Flowering mechanism: a case study in blueberry

The highbush blueberry (2n = 4x = 48), a prominent cultivated member of the *Vaccinium* fruit crop family, has a rather substantial chilling requirement, typically surpassing 800 chilling units, that must be met to initiate dormancy release during spring (Song et al., 2011; Edger et al., 2022). Over the course of past decades, extensive investigations have been carried out to reveal the flowering mechanism (Song et al., 2023). A summary of these blueberry studies can serve as an illustrative example, providing insights into the network of factors that underlie flowering mechanisms in woody plants.

### 4.1 *VcFT* is a major floral initiator

**Figure 3:**
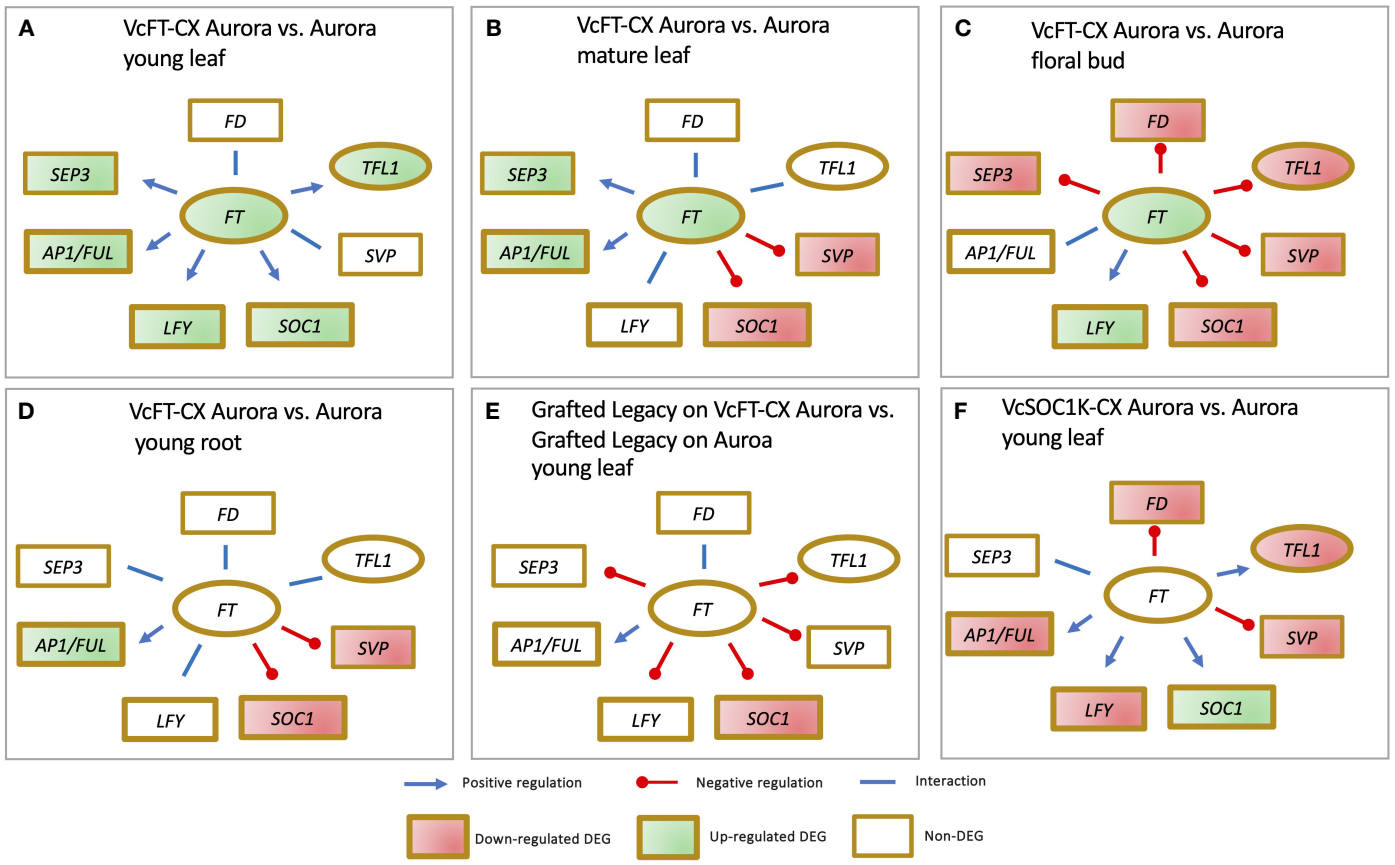
RNA-sequencing data reveals impact of VcFT-CX **(A-E)** and VcSOC1K-CX **(F)** on major flowering pathway genes in different tissues.

The impact of VcFT-CX is evident across different plant species. In tobacco (*Nicotiana tabacum*) and petunia (*Petunia* x *hybrid*), VcFT-CX not only induced early flowering but also led to plant dwarfing (Song et al., 2013b). Similarly, under nonchilling conditions, the northern highbush blueberry cultivar Aurora exhibited precocious flowering as a result of VcFT-CX (Song et al., 2013b). At the transcript level, VcFT-CX triggered a substantial increase in *VcFT* expression in leaves and nonchilled floral buds, while its effect on young roots was not significant (Walworth et al., 2016; Song et al., 2019; Song et al., 2023). Notably, VcFT-CX displayed distinct effects on various tissues and developmental stages (Walworth et al., 2016; Song et al., 2019; Song et al., 2023). In young leaves, it upregulated the expressions of *VcAP1/VcFUL*, blueberry *SEPALLATA* (*VcSEP*), *VcLFY*, *VcSOC1*, and *VcTFL1*, with no significant changes in *VcSVP* and *VcFD* (**Figure 3A**) (Walworth et al., 2016). In mature leaves, VcFT-CX enhanced *VcAP1/VcFUL* and *VcSEP* expressions, while repressing *VcSOC1* and *VcSVP*, and it had minimal impact on *VcLFY*, *VcFD*, and *VcTFL1* (**Figure 3B**) (Song et al., 2023). In nonchilled flower buds, VcFT-CX upregulated *VcLFY* expression, downregulated *VcSEP*, *VcSOC1*, *VcSVP*, *VcFD*, and *VcTFL1*, while *VcAP1/VcFUL* expression remained relatively unaffected (**Figure 3C**) (Song et al., 2023). In young roots, *VcAP1/VcFUL* expression increased, while *VcSOC1* and *VcSVP* decreased; *VcSEP*, *VcLFY*, *VcFD*, and *VcTFL1* showed no significant changes (**Figure 3D**) (Song et al., 2019). Key takeaways from the analysis of VcFT-CX tissues include: 1) Varied responses of major flowering pathway genes (*e.g*., *VcSEP3*, *VcSOC1*, and *VcSVP*) to VcFT-CX across tissues and developmental stages, with a consistent promotion of *VcLFY* and *VcAP1/VcFUL* expression; 2) Enhanced expressions of *VcAP1/VcFUL* and *VcSEP* in leaf tissues due to VcFT-CX, indicating the potential role of these genes in floral initiation, while *VcFD* and *VcTFL1* seem less involved in promoting floral initiation; 3) Repression of *VcFD* and *VcTFL1* expressions in nonchilled floral buds by VcFT-CX; and 4) Likely pivotal roles of *VcSOC1* and *VcSVP* in the activation of floral buds under chilling conditions.

Moreover, as rootstocks, VcFT-CX produced signals in leaves that were effectively conveyed through transgrafting to nontransgenic scions (cv. Legacy), yielding a distinctive enhancement in floral bud formation (Song et al., 2019). Intriguingly, at the transcript level, VcFT-CX in rootstocks did not trigger differential expression of *VcFT*, *VcFD*, *VcTFL1*, *VcAP1/VcFUL*, *VcLFY*, *VcSVP*, and *VcSEP3* in the grafted nontransgenic scions. Interestingly, expression of VcSOC1 was significantly downregulated (**Figure 3E**). Notably, there is an instance where none of the identified major flowering pathway genes (*e.g., VcFT*, *VcAP1/VcFUL, VcSOC1*, and *VcL*FY) appear to solely account for the promoted floral bud formation, indicating that an escalated *VcFT* expression is not always the sole requirement for initiating flowering (Song et al., 2019). As for the potential long-distance florigenic signals inducing from VcFT-CX, their precise nature remains to be discerned from candidates like *VcFT* protein/mRNA, cytokinin, or other hormonal factors (Gao et al., 2016; Walworth et al., 2016; Song et al., 2019).

### 4.2 *VcSOC1* is a major floral activator

**Figure 4:**
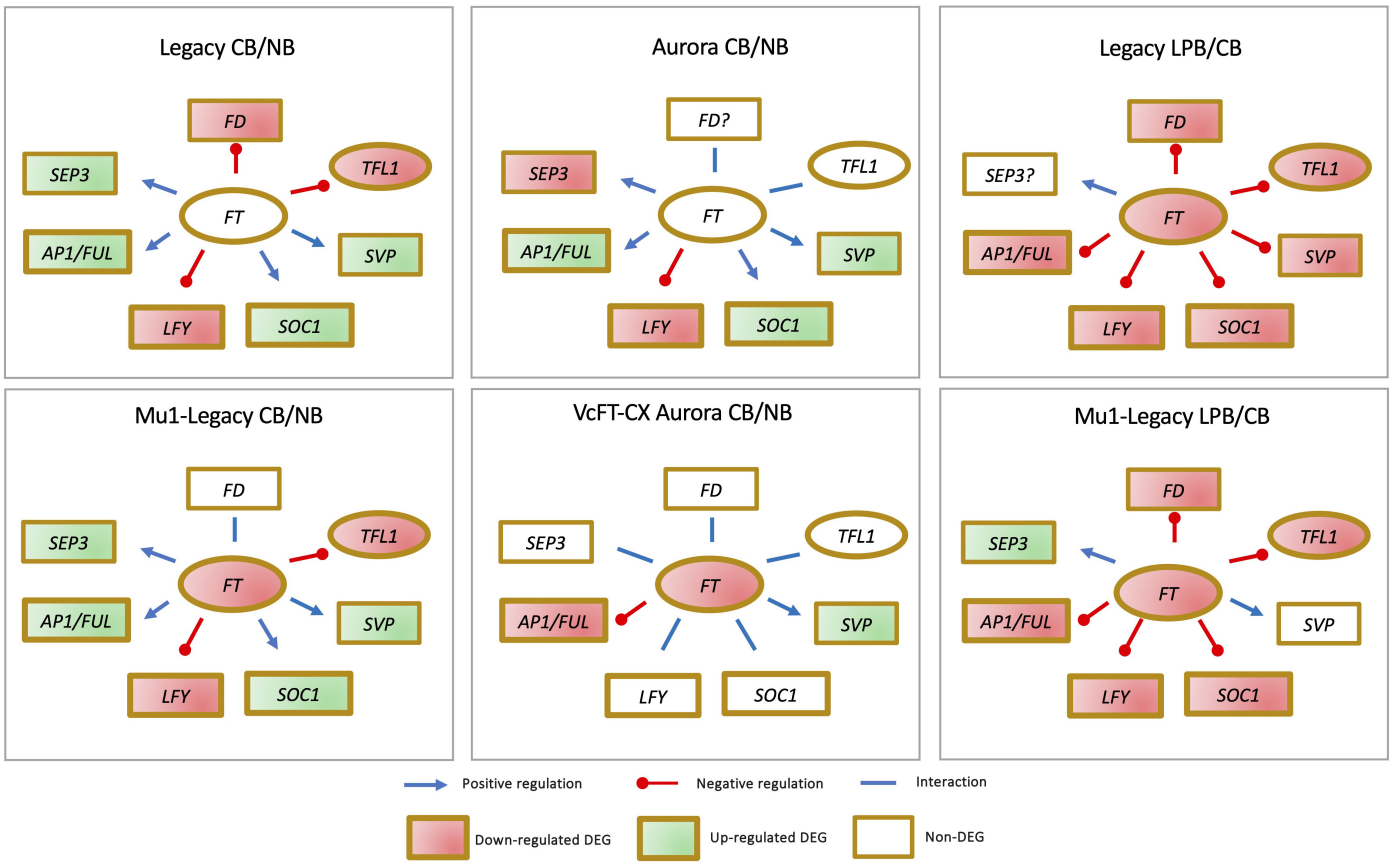
Distinctive gene expression patterns observed in the comparisons between fully chilled floral buds (CB) and nonchilled floral buds (NB), as well as late pink bud (LPB) versus CB, across different genotypes.

Comparative analyses of floral buds have been conducted for four genotypes, including a nontransgenic northern highbush variety Aurora, a VcFT-CX transgenic ‘Aurora’, a nontransgenic southern highbush variety Legacy, and a transgenic Legacy mutant (Mu1-Legacy) (**Figure 4**) (Song and Chen, 2018b; Song and Walworth, 2018; Song et al., 2023). Among these four comparisons: 1) *VcFT* expression demonstrated either negligible differential expression in nontransgenic cultivars or downregulation in the two transgenic genotypes, suggesting that *VcFT* may not a primary target of chilling accumulation for bud break; 2) Expression of *VcLFY*, *VcTFL1*, and *VcFD* remained either suppressed or constant post full chilling; 3) *VcAP1/VcFUL* expression increased in three genotypes and decreased in VcFT-CX ‘Aurora’; and 4) *VcSOC1* expression was upregulated in three genotypes and exhibited no significant differential expression in VcFT-CX ‘Aurora’, while VcSVP expression was elevated in all four genotypes (**Figure 4**). Collectively, *VcSOC1* and *VcSVP* played key roles in chilling requirement-mediated floral activation. Interestingly, in transcriptomic comparisons between late pink buds and fully chilled stages for two genotypes, expressions of *VcFT*, *VcFD*, *VcTFL1*, *VcAP1/VcFUL*, *VcLFY*, and *VcSOC1* were uniformly repressed in late pink buds (**Figure 4**) (Song and Chen, 2018b).

Further evidence supporting *VcSOC1* as a significant floral activator is the fact that the constitutive expression of the K domain of VcSOC1 (VcSOC1K-CX) led to the flowering of transgenic ‘Aurora’ plants under nonchilling conditions, a condition where nontransgenic ‘Aurora’ plants remained unable to flower (Song and Chen, 2018a). SOC1 is classified as a type-II plant-specific MIKC protein, characterized by its conserved MADS (M-), intervening (I), keratin-like (K-), and C-terminal (C-) domains (Theissen et al., 1996). The K domain is instrumental in facilitating interactions among various MADS box genes. Remarkably, VcSOC1K-CX has also demonstrated the ability to accelerate flowering in tobacco and maize (Song et al., 2013a; Song and Han, 2021). In blueberry, the promotion of flowering through VcSOC1K-CX was associated by the increased expression of *VcSOC1*, which in turn led to the repression of *VcFT*, *VcFD*, *VcTFL1*, *VcAP1*/*VcFU*L, *VcLFY*, and *VcSVP*, offering another piece of evidence that the elevation of VcFT expression is not always a prerequisite for flowering promotion in ‘Aurora’ (**Figure 3F**) (Song and Chen, 2018a).

**Figure 5:**
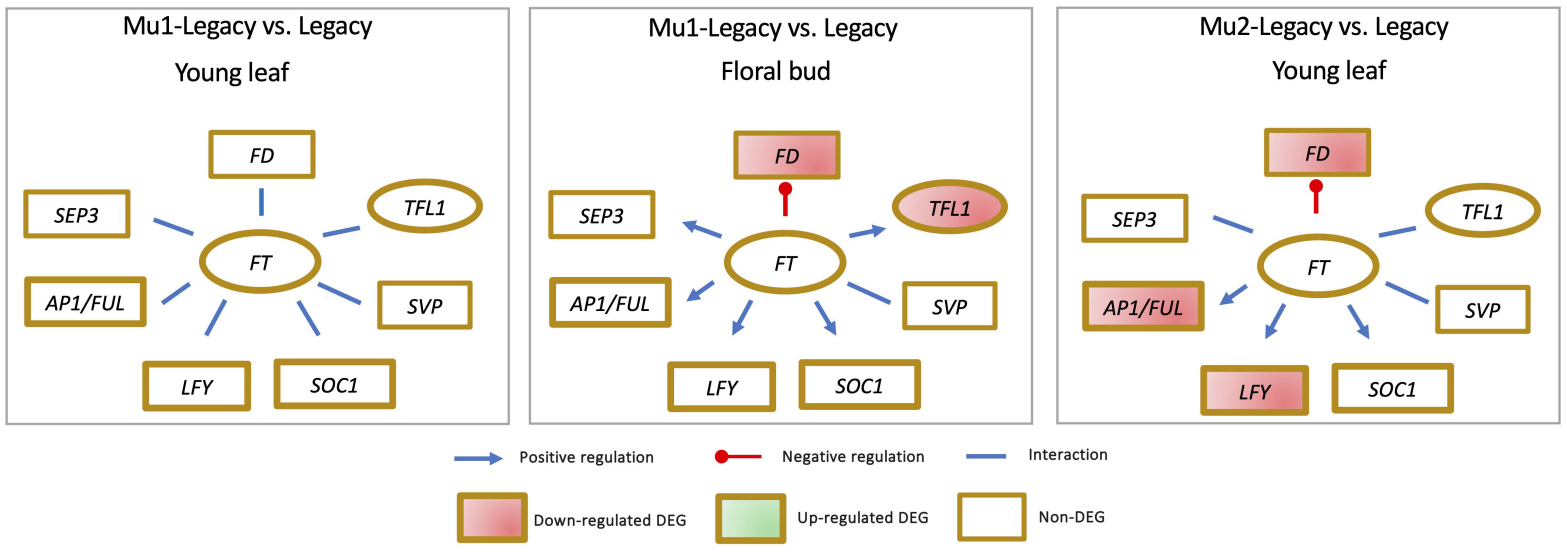
The identification of differential expression of genes (DEGs) in blueberry mutants indicates that elevated VcFT expression is not always a prerequisite for achieving precocious or early flowering.

While the expression of *VcSOC1* is indeed crucial for CR-mediated floral activation in blueberry, it is important to note that *VcSOC1* does not always play an obligatory role in floral bud activation. An intriguing instance is presented by the Mu1-Legacy genotype, which carries an overexpressed blueberry *DWARF AND DELAYING FLOWERING 1* gene (*VcDDF1*), allowing it to flower under nonchilling conditions, a feat nontransgenic ‘Legacy’ plants could not achieve (Song and Walworth, 2018). Remarkably, in this scenario, none of the major genes—*VcSOC1*, *VcFT*, *VcAP1/VcFUL*, *VcLFY*, *VcSVP*, and *VcSEP3*—displayed discernible differential expression in both young leaves and floral buds (**Figure 5**) (Song and Walworth, 2018; Lin et al., 2019).

As of now, functional *FLC*-like candidates have not been definitively identified, despite the presence of orthologues of many other vernalization pathway genes from Arabidopsis in blueberry (Walworth et al., 2016). Notably, the intriguing case of an apple *FLC3*-like gene stands out, as its constitutive expression surprisingly promotes flowering instead of causing the expected delay (Zong et al., 2019). Although there have been studies examining the effects of chilling accumulation on the expression of flowering pathway genes, the impact of warm accumulation on the activation of fully chilled floral buds remains an area yet to be thoroughly explored. In light of the available literature, it is apparent that among the genes within the flowering pathway, *VcSOC1* plays a pivotal role as a major floral activator.

### 4.3 A *VcFT/VcSOC1* regulatory module in blueberry flowering

In general, the expression of *FT* within leaves is significantly influenced by light conditions, whereas *SOC1* expression is modulated in response to temperature changes. Notably, *VcFT* attains its peak expression in floral buds, while *VcSOC1* reaches its highest expression level in leaves (Walworth et al., 2016). Recently, a regulatory framework centered on the ratio of FT-to-SOC1 expression (*VcFT/VcSOC1*) has been proposed, providing a valuable lens through which to comprehend the processes of floral initiation and activation. According to this model, an elevated *VcFT/VcSOC1* ratio in leaves serves to stimulate floral initiation, while heightened *VcSOC1* expression can lead to early flowering. Within flower buds, the *VcFT/VcSOC1* ratios often decline in chilled buds due to the increasing *VcSOC1* expression during chilling accumulation, whereas emerging flower buds exhibit rising *VcFT/VcSOC1* ratios due to the more rapid decline of *VcSOC1* expression relative to *VcFT* (Song et al., 2023). This principle is further bolstered by observations of reduced *FT/SOC1* ratios in polar buds during chilling accumulation, marked by an upsurge in *SOC1* expression alongside neutral *FT* levels (Gómez-Soto et al., 2021). Nonetheless, it’s important to acknowledge that this *VcFT/VcSOC1* ratio might not be universally applicable to all tested blueberry genotypes; for instance, the altered flowering pattern in the Legacy-mutant1 was not attributable to major flowering pathway genes (Song and Walworth, 2018). Collectively, this *FT/SOC1* ratio emerges as a potential determinant of both leaf-based floral initiation and bud-based floral activation, given that these genes hold pivotal roles as integrators within the flowering pathway.

### 4.4 Other regulatory genes for floral initiation or activation beyond flowering pathway genes

In addition to the well-defined flowering pathway genes, there exists a range of genes from other pathways that exert influence over floral initiation and activation. A notable instance, as discussed earlier, is the altered floral initiation and activation process observed in Mu1-Legacy, wherein neither *VcFT* nor *VcSOC1* played a role (**Figure 5**). Upon scrutinizing the transcriptomic analysis of blueberry flowering pathway genes, it becomes evident that numerous genes from hormone and sugar pathways are intricately linked to floral bud initiation or activation, whether through direct or indirect means (Gao et al., 2016; Lin et al., 2019; Song et al., 2019). This underlies the fact that hormones and sugar pathway genes, in conjunction with the flowering pathway genes, likely participate in a coordinated manner to regulate the process of flowering (Izawa, 2021).

## 5 Conclusion

The well-established genetic framework governing flowering pathways in Arabidopsis has served as a cornerstone for unraveling the intricate mechanisms operating in other plants. Woody plants, however, have developed notably complex flowering pathways compared to Arabidopsis, although some key flowering pathway genes maintain largely conserved roles (**Table 1**, **Figure 2**). The CO-FT module within the photoperiod pathway, which is crucial in Arabidopsis, appears to be considerably conserved in woody plants, although the functions of *CO* or *COL* orthologues in this context warrant further investigation. The miR156-SPL module of the age pathway exhibits conservation across all plant species, notwithstanding the varied roles of *SPL*s in woody plants. In the GA pathway, the interactions involving GA and DELLA factors demand deeper exploration in both Arabidopsis and woody plants due to their extensive influence on both floral initiation and activation. The *FLC*-mediated vernalization pathway, a central mechanism in Arabidopsis, exhibits the least conservation in woody plants, where effective chilling is requisite to initiate flowering. Nonetheless, it’s noteworthy that MADS-box genes play significant roles in floral activation. In essence, among the individual flowering pathway genes, *FT* and its orthologues serve as pivotal floral initiators, while *SOC1* and its orthologs stand as the principal floral activators. Remarkably, this pattern remains highly conserved across plant species.

## Author contributions

G-qS: Conceptualization, Writing – original draft, Writing – review & editing. ZL: Writing – review & editing. G-yZ: Writing – review & editing.
